# Phenotypic and Genotypic Characterization of Antimicrobial Resistance in *Helicobacter pylori*: Association of *23S rRNA*, *gyrA*, and *rdxA* Gene Mutations With Resistance Patterns

**DOI:** 10.1155/ijm/6456370

**Published:** 2026-07-06

**Authors:** Amal F. Makled, Hany A. Abdelaziz, Dina M. Allam, Mona F. Salama, Nashwa N. Khamis, Asmaa S. Sleem

**Affiliations:** ^1^ Department of Medical Microbiology and Immunology, Faculty of Medicine, Menoufia University, Shebin al Kom, Egypt, menofia.edu.eg; ^2^ Department of Internal Medicine, Gastroenterology and Hepatology, Faculty of Medicine, Menoufia University, Shebin al Kom, Egypt, menofia.edu.eg; ^3^ Department of Pathology, Faculty of Medicine, Menoufia University, Shebin al Kom, Egypt, menofia.edu.eg; ^4^ Department of Clinical Pathology, Faculty of Medicine, Menoufia University, Shebin al Kom, Egypt, menofia.edu.eg

**Keywords:** antimicrobial resistance, *Helicobacter pylori*, mutations, sequencing

## Abstract

**Background:**

Antibiotic‐resistant *Helicobacter pylori* pose a significant global health challenge. This Egyptian study is aimed at characterizing resistant *H. pylori* isolates and investigate the association between *23S rRNA*, *gyrA*, and *rdxA* mutations and phenotypic profiles.

**Patients and Methods:**

Gastric biopsies collected in the internal medicine endoscopy unit were *H. pylori* tested by rapid urease, histopathology, and culture confirmed by *ureC* PCR. Antimicrobial susceptibility to clarithromycin, levofloxacin, and metronidazole was evaluated by disk diffusion and agar dilution methods. Sanger sequencing was employed to detect *23S rRNA*, *gyrA*, and *rdxA* mutations.

**Results:**

Among 81 cultivable *H. pylori* isolates, bacillary 37/81 (45.7%), coccobacillary 30/81 (37.1%), and coccoid 14/81 (17.2%) forms were seen. Agar dilution confirmed resistance to metronidazole, clarithromycin, and levofloxacin by 72.8%, 23.5%, and 9.9%, respectively. Dual resistance was confirmed by 22.3% versus 2.5% triple resistance. Sequencing results of eight resistant isolates revealed *23S rRNA* mutations (A2143G, A2142G; 5/8), whereas *gyrA* mutations included D91N (2/8), D91G, and N87K (1/8 each). A 200‐bp *rdx* deletion was detected (3/8) along with D59N (2/8), R131K, A68N, and truncation mutations (each 1/8). Coccoid (4/8) and coccobacillary (2/8) *H. pylori* forms mixed with gastric microbiota (6/8), which also demonstrated dual (4/8) or triple (2/8) antibiotic resistance, and showed an association with the A2143G and A2142G mutations plus 200‐bp deletion in the *rdxA* gene.

**Conclusion:**

Integrating molecular genotyping with phenotypic diagnostics may improve *H. pylori* resistance characterization. Findings from this limited number of sequenced resistant isolates suggest a possible association between *23S rRNA*, *gyrA*, and *rdxA* mutations and virulent, resistant, coccoid‐microbiota mixed strains.

## 1. Introduction

*Helicobacter pylori* is helical, gram‐negative bacteria that infect nearly half of the world population. In Egypt, the prevalence of *H. pylori* infection exceeds 50% [1]. Although most infections remain asymptomatic, immune‐evasive strains are capable of causing peptic ulcers and gastric malignancies [2].

The rising *H. pylori* antimicrobial resistance poses a significant threat to the efficacy of current therapeutic regimens. For instance, clarithromycin resistance can reduce the effectiveness of the standard proton pump inhibitor (PPI)/amoxicillin–based triple therapy by up to 70%. Similarly, resistance to levofloxacin is associated with failure of second‐line or salvage therapies, emphasizing the need for alternative treatment options [3].

The molecular basis of antibiotic resistance in *H. pylori* is primarily attributed to genetic mutations that either alter the antibiotic target site or reduce intracellular drug concentration. *H. pylori* antimicrobial resistance is best identified through molecular diagnostics [4]. Peptidyl transferase region mutations such as A2143G, A2142G, and A2142C within domain V of *23S rRNA* gene are responsible for over 80% clarithromycin resistance. Levofloxacin resistance is commonly associated with point mutations in *gyrA* and *gyrB* genes of quinolone resistance‐determining region (QRDR) [5]. Moreover, *rdxA* and, less commonly, *frxA* mutations have been implicated in metronidazole resistance [6].

Antibiotic resistance patterns are often shaped by geographic antibiotic usage protocols, reflecting differences in host, microbial, and environmental factors. These variations underscore the diagnostic and therapeutic challenges faced in clinical practice and highlight the need for region‐specific resistance profiling and management protocols [7].

Therefore, the aim of this study was to evaluate antimicrobial resistance among *H. pylori* gastric isolates using both phenotypic and molecular methods to correlate resistance‐associated mutations in the *23S rRNA*, *gyrA*, and *rdxA* genes with observed phenotypic patterns.

## 2. Patients and Methods

### 2.1. Study Design, Patient Selection, and Ethical Considerations

This analytical study was conducted in the Medical Microbiology and Immunology Department, Faculty of Medicine in collaboration with Internal Medicine Endoscopy Unit, Menoufia University hospitals, Egypt—from July 2023 to December 2024. A total of 240 adult patients presenting with dyspeptic symptoms and clinically suspected *H*. *pylori* infection were enrolled. Exclusion criteria included recent sclerotherapy or band ligation for esophageal varices, coagulation disorders, or receipt of *H. pylori* therapy within the last 4 weeks [8]. The study protocol was approved by the Menoufia University Ethics Committee (IRB: 7/2023MICR20), and written informed consent was obtained from all participants.

### 2.2. Specimen Collection and Processing

Upper gastrointestinal endoscopy was performed by a consultant gastroenterologist. From each participant, six gastric mucosal biopsies were obtained (three from the antrum and three from the corpus) using sterile, single‐use biopsy forceps. The specimens were allocated into three sets: The first set was used for the rapid urease test (RUT), the second was fixed in 10% buffered formalin for histopathological examination, and the third was placed in brain heart infusion (BHI) broth supplemented with glycerol for microbiological analysis [9].

### 2.3. Isolation of *H. pylori*

Biopsies were homogenized and cultured on selective (Columbia agar with 8% human blood and DENT supplement) and nonselective blood agar. Plates were incubated for 3–7 days under microaerophilic conditions (5% O_2_, 10% CO_2_) using a Gaspak jar and Compagyn microaerophilic sachets [10]. Presumptive *H. pylori* colonies appeared as small, translucent, whitish growths identified by Gram staining (gram‐negative helical bacilli) showing positive oxidase, catalase, and urease tests [11]. Isolates were confirmed using *ureC* PCR detection [12] as shown in Table 1.

**Table 1:** Primers used in molecular analysis of *Helicobacter pylori* isolates and the corresponding thermal cycling conditions for target genes.

Gene	Primer sequence (5 ^′^ →3 ^′^)	Thermal cycling conditions					Size (bp)	Reference
		Initial denaturation (°C/time)	Denaturation (°C/time)	Annealing (°C/time)	Cycles	Final extension (°C/minute)		
***ureC***	F: GGATAAGCTTTTAGGGGTGTTAGG	94/4 min	94/1 min	56/45 s	30	72/10	294	[13]
	R: GCTTACTTTCTAACACTAACGCGC							
***23S rRNA***	F: CCACAGCGATGTGGTCTCAG	96/5 min	96/30 sec	60/25 s	35	72/5	425	[14]
	R: CTCCATAAGAGCCAAAGCCC							
***gyrA***	F: AGCTTATTCCATGAGCGTGA	96/5 min	96/30 min	52/25 s	35	72/5	582	[14]
	R: TCAGGCCCTTTGACAAATTC							
***rdxA***	F: AATTTGAGCATGGGGCAGA	94/5 min	94/1 min	55/1 min	30	72/10	850	[15]
	R: GAAACGCTTGAAAACACCCCT							

*Note:* Primer extension at 72°C for 1 min was used for all target genes preceding the final extension step.

To detect coexisting microbiota, biopsies were also cultured on nonselective blood agar, and non‐*H. pylori* colonies were preliminarily identified by Gram stain and basic biochemical tests [16].

### 2.4. Antimicrobial Susceptibility Testing

Antimicrobial susceptibility of confirmed *H. pylori* isolates was assessed using two phenotypic methods:

A. Disk diffusion (Kirby–Bauer) method: Susceptibility to clarithromycin, levofloxacin, and metronidazole was assessed using the Kirby–Bauer disk diffusion method. The inhibition zone diameter breakpoints were calculated corresponding to the European Committee on Antimicrobial Susceptibility Testing break points [17, 18].
B. Agar dilution method: Minimum inhibitory concentrations (MICs) for clarithromycin, levofloxacin, and metronidazole were determined using the agar dilution technique and interpreted based on the EUCAST, 2022 recommended MIC break points [17].

### 2.5. Molecular Detection of Clarithromycin, Levofloxacin, and Metronidazole Resistance Determinants

#### 2.5.1. Conventional PCR Amplification

It was performed on *H. pylori* isolates that exhibited phenotypic resistance to clarithromycin, levofloxacin, or metronidazole, as determined by the agar dilution method. Target genes included *23S rRNA*, *gyrA*, and *rdxA*, with amplification protocols optimized to confirm gene presence. Amplification data are summarized in Table 1.

#### 2.5.2. Sanger Sequencing

It was performed to identify *23S rRNA*, *gyrA*, and *rdxA* gene mutations for eight representative purposively selected *H. pylori* to include different endoscopic findings and diverse antimicrobial resistance phenotypes with varying MIC levels. Following PCR amplification and DNA purification, sequencing was conducted according to the protocol described by Daniels [19]. The thermal cycling protocol consisted of an initial denaturation at 96°C for 1 min, followed by 25 cycles of 96°C for 10 s, 58°C for 5 s, and 60°C for 4 min, with a final hold at 4°C. Sequencing was carried out using the ABI PRISM 310 Genetic Analyzer (Applied Biosystems, Foster City, California, United States).

#### 2.5.3. Sequence Analysis

Sequencing data were electronically retrieved and analyzed using Chromas (v2.6.6) and FinchTV (v1.4.0). Comparative alignment with the reference strain *H. pylori* 26695 (GenBank Accession Number CP003904), was performed using MEGA 11 (v11.0.10) to identify point mutations associated with antimicrobial resistance [20].

### 2.6. Statistical Analysis

Data were collected, tabulated, and analyzed using the Statistical Package for Social Sciences (SPSS), Version 26 (SPSS Inc., Chicago, Illinois, United States). Statistical tests included the chi‐square (*χ* ^2^) test, Fisher′s exact test, and calculation of diagnostic accuracy parameters. A *p* value < 0.05 was considered statistically significant.

## 3. Results

**Figure 1:**
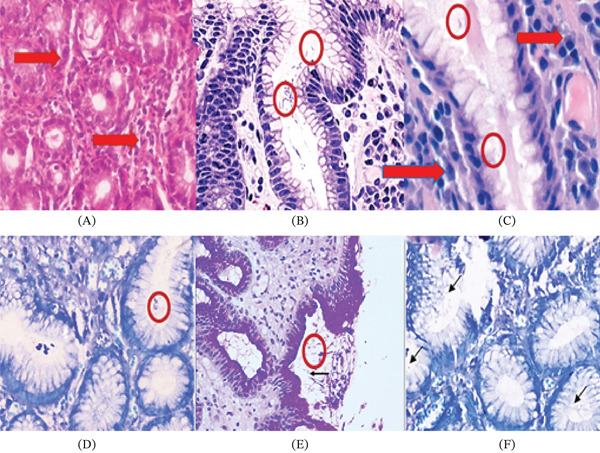
Microscopic forms and colonization patterns of *Helicobacter pylori* in gastric biopsies These figures show the microscopic picture of *H. pylori* gastritis, highlighting the various morphological forms of *H. pylori* (bacillus, coccobacillus, and coccoid) in different *H. pylori* **–**infected gastric biopsies using hematoxylin–eosin (H&E) and Giemsa stains, highlighting the bacterial adaptability and its role in gastric mucosal inflammation. Panel (A) shows active *H. pylori* gastritis with lymphocytes and plasma cells infiltrating the lamina propria, with focal clusters of neutrophils infiltrating the gastric glands (red arrows) (H&E × 200). Panels (B and C) show chronic inflammatory cellular infiltrate in the form of lymphocytes and plasma cells in the lamina propria (red arrows), and *H. pylori* bacilli carpeting luminal surface of mucous glands (red circles) (H&E × 400). Panels (D, E, and F) show different grades of gastritis with inflammatory infiltrates in the lamina propria, infected *H pylori* bacilli (red circles) and *H pylori* coccoid form (black arrow) (Giemsa stain × 400).

This study analyzed 81 *H*. *pylori* isolates obtained from 240 patients, classified by endoscopic findings into nonulcer dyspepsia (NUD) (77.9%), peptic ulcer disease (PUD) (19.6%), and gastric cancer (GC) (2.5%) (Figure S1). To detect *H. pylori*, we used RUT, which showed the highest detection rate (65%), followed by histopathology (53.7%) with peak detection observed in PUD patients (76.6%). Among 129 histopathological positive cases, three microscopic forms of *H. pylori* were observed, the coccobacillary form was predominated 45/129 (44.9%), bacillary form 58/129 (34.8%), whereas the coccoid form was observed in 20.3% (26/129) of cases, including GC patients (Figure 1). *H. pylori* were identified based on their localization along the gastric epithelium, the characteristic histological features of *H. pylori*–associated gastritis (chronic active inflammation with neutrophilic activity and lymphoid aggregates) confirmed by Giemsa stain. These criteria are commonly used to differentiate *H. pylori* from other gastric microbiota [21].

Culture confirmed *H. pylori* in 33.7% of cases, with the highest recovery in PUD (55.3%), in contrast to only one GC case, suggesting potential challenges in culturing more virulent strains (Table 2). Nonselective media also revealed diverse gastric microbiota, mainly gram‐negative bacteria, especially with NUD and PUD patients (Figure S2). Among culture‐positive cases, anemia (33.3%) and weight loss (40.7%) were significantly more frequent.

**Table 2:** Diagnostic methods and microscopic forms of gastric *H. pylori* according to endoscopic findings.

**Different diagnostic methods**	**Total**		**Endoscopic groups**						**χ** ^2∗^	**p** **value**
	**(** **N** = 240**)**		**NUD**		**PUD**		**GC**			
			**(** **N** = 187**)**		**(** **N** = 47**)**		**(** **N** = 6**)**			
	**N**	**%**	**N**	**%**	**N**	**%**	**N**	**%**		

**Rapid urease test**										
Positive	156	65.0	21	64.7	29	61.7	6	100.0	3.46	0.177
Negative	84	35.0	66	35.3	18	38.3	—	—		
**Culture growth**									**FE**	
Positive	81	33.7	54	28.9	26	55.3	1	16.6	12.55	**0.002**
Negative	159	66.3	133	71.1	21	44.7	5	83.4		(S)
**Histopathology**									**FE**	
Positive	129	53.7	89	47.6	36	76.6	4	66.7		**0.001**
Negative	111	46.3	98	52.4	11	23.4	2	33.3	13.12	(S)

**Microscopic forms of *H. pylori* in histopathology-positive cases (** **N** = 129**)**	**Total**		**NUD**		**PUD**		**GC**		**χ** ^2∗^	**p** **value**
	**(** **N** = 129**)**		**(** **N** = 89**)**		**(** **N** = 36**)**		**(** **N** = 4**)**			
	**N**	**%**	**N**	**%**	**N**	**%**	**N**	**%**		

Bacilli	45	34.8	36	40.4	9	25	—	—		
Coccobacilli	58	44.9	49	55.1	9	25	—	—	49.59	**< 0.001**
Coccoid	26	20.3	4	4.5	18	50	4	100		**(HS)**

*Note:* *χ* ^2^: chi‐square test comparing nonulcer dyspepsia (NUD), peptic ulcer disease (PUD), and gastric cancer (GC) groups. S: statistically significant (*p* < 0.05). HS: highly significant (*p* < 0.001). The asterisks in Table 2 denote that these values are highly significant. Bold values indicate the total number.

Abbreviation: FE, Fisher′s exact.

A comparative assessment of disk diffusion results using the agar dilution method as the reference standard was conducted to evaluate *H. pylori* resistance to clarithromycin, levofloxacin, and metronidazole (Table 3). Agar dilution revealed high resistance rates to metronidazole (72.8%), whereas susceptibility remained high for levofloxacin (90.2%) and clarithromycin (76.5%). The distribution of resistance was as follows: mono‐resistance in 54.3%, dual resistance in 22.3%, and triple resistance in 2.5% of isolates, with metronidazole most frequently involved among all patterns. Resistance patterns were associated with bacterial morphology: Coccobacillary and coccoid forms recorded strong association with dual and triple resistance.

**Table 3:** Diagnostic performance of disk diffusion versus agar dilution methods for *H. pylori* antibiotic susceptibility testing (*N* = 81).

Antibiotic phenotypic susceptibility tests (*N* = 81)				Accuracy	Sensitivity	Specificity	PPV	NPV
**Clarithromycin (disk diffusion method)**	**Clarithromycin (agar dilution method) Range (0.125–16 *μ*g/mL)**		**Total** **N** **(%)**					
	**R**	**S**						
	N (%)	N (%)						
Resistant (R)	18	12	30 (63)					
Sensitive (S)	1	50	51 (37)					
Total	19 (23.5)	62 (76.5)	81	84%	94.7%	80.6%	60.0%	98.0%

**Levofloxacin (disk diffusion method)**	**Levofloxacin (agar dilution method) range (0.125–4 *μ*g/mL)**		Total *N* (%)					
	R	S						
	*N* (%)	*N* (%)						
Resistant (R)	8	2	10 (12.3)					
Sensitive (S)	0	71	71 (87.7)					
Total	8 (9.9)	73 (90.1)	81	97.5%	100%	97.3%	80%	100%

**Metronidazole (disk diffusion method)**	**Metronidazole (agar dilution method) range (1–64 *μ*g/ml)**		Total *N* (%)					
	R	S						
	*N* (%)	*N* (%)						
Resistant (R)	59	11	70 (86.4)					
Sensitive (S)	0	11	11 (13.6)					
Total	59 (72.8)	22 (27.2)	81	86.4%	100%	50.0%	84.3%	100%

*Note:* Sensitivity, specificity, accuracy, PPV (positive predictive value), and NPV (negative predictive value) were calculated using agar dilution as the reference standard.

We focused on *H. pylori* resistance against clarithromycin and metronidazole, as these are reported to be commonly used therapeutic options in our region. In addition, it was also important to evaluate and assess resistance to levofloxacin, since it has recently been incorporated into newer *H. pylori* eradication regimens in Egypt for patients with recurrent infection or having contraindications to the conventional therapies [22].

There was no significant difference between the various resistance patterns and the sensitive *H. pylori* isolates to the three tested antibiotics (clarithromycin, levofloxacin, and metronidazole) regarding sociodemographic characteristics and clinical data. However, high infection recurrence showed a significant association with dual and triple resistance patterns, whereas it was not observed among patients harboring sensitive isolates.

**Figure 2:**
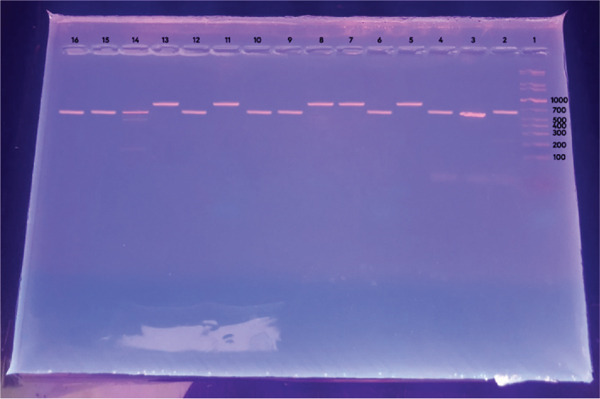
Agarose gel electrophoresis of amplified *H. pylori rdxA* gene using conventional PCR showing wild and mutant isolates. Wild type (850 bp): Lanes 5, 7, 8, 11, and 13; 200‐bp deletion mutant (650 bp): Lanes 2, 4, 6, 9, 10, 12, 14, and 16; Lane 1: 100–1000‐bp DNA ladder.

Resistance‐associated genes (*23S rRNA*, *gyrA*, and *rdxA*) were further analyzed. A distinct 200‐bp deletion in the *rdxA* gene was detected in 47.5% (28/59) of metronidazole‐resistant isolates, whereas metronidazole‐sensitive isolates showed normal band sizes, suggesting an association between the 200‐bp deletion and resistance. No abnormal bands were observed for *gyrA* or *23S rRNA* (Figure 2).

Sequencing of targeted resistance genes was conducted on eight representative isolates. Mutations in the *23S rRNA* gene were found in 5/8 isolates (62.5%): A2143G (*n* = 2), A2142G (*n* = 2), plus dual A2142G + A2143G mutation (*n* = 1). *gyrA* mutations were identified in four levofloxacin‐resistant isolates: D91N (*n* = 2), D91G (*n* = 1), and N87K (*n* = 1). *rdxA* mutations were detected in metronidazole‐resistant isolates (4/8), including D59N (*n* = 2), R131K (*n* = 1), and truncating mutations (*n* = 1). A single metronidazole‐sensitive isolate exhibited an A68N variant of uncertain significance.

Now, among the 59 metronidazole‐resistant isolates, 28 (47.5%) exhibited the characteristic 200‐bp *rdxA* deletion, which was identified by PCR analysis. Because this deletion had already been confirmed, sequencing of the *rdxA* gene was not performed for these isolates. Representative resistant isolates were subsequently selected for sequencing based on predefined selection criteria to investigate mutations in the studied resistance‐associated genes. Accordingly, some of the selected isolates carried the 200‐bp *rdxA* deletion as determined by PCR and were included to explore whether this deletion coexisted with mutations in other resistance‐related genes. Although some sequenced isolates showed identifiable mutations, additional mutations or alternative resistance mechanisms may also exist in nonsequenced isolates (Table 4). Table 4 summarizes the mutations detected exclusively in the eight representative isolates selected for sequencing and does not reflect the mutation profiles of all resistant isolates.

**Table 4:** Correlation between antibiotic susceptibility by agar dilution method and studied genes associated mutations in sequenced *H. pylori* isolates (*N* = 8).

Antibiotic/resistance gene	Antibiotic susceptibility of selected isolates (*N* = 8)		Key mutations in resistant group		Key mutations in sensitive group	
	Resistant	Sensitive	Mutation	(%)	Mutation	(%)
**Clarithromycin/*23S rRNA* gene**	5	3	A2143G	40	A2142G	33.3
			A2142G	20		
			(A2143G + A2142G)	20	No mutation	66.7
			No mutation	20		

**Levofloxacin/*gyrA* gene**	4	4	D91N	50	No mutation	100
			D91G	25		
			N87K	25		

**Metronidazole/*rdxA* gene**	7	1	200‐bp deletion	42.9	A68N	100
			D59N	28.5		
			R131K	14.3		
			Truncation mutation	14.3		

*Note:* (*%*) = percentage of each mutation in each group (resistant isolates and sensitive isolates) for each antibiotic *. rdxA*, *23S rRNA*, and *gyrA* combined mutations were detected in three isolates. *rdxA* and *23S rRNA* combined mutations were detected in two isolates. *rdxA* and *gyrA* combined mutations were detected in only one isolate. Single *rdxA* mutation was detected in two isolates.

Risk analysis showed that the A2143G mutation in the *23S rRNA* gene was detected exclusively among clarithromycin‐resistant isolates suggesting a possible association with resistance, whereas A2142G showed a weaker association with resistance. Most isolates lacking these mutations were susceptible to clarithromycin, although a few resistant isolates were also observed. Similarly, *gyrA* mutations (N87K, D91N, and D91G) were observed in levofloxacin‐resistant isolates, whereas most isolates without *gyrA* mutations were susceptible. For metronidazole, mutations D59N and a 200‐bp deletion in *rdxA* were mainly detected in resistant isolates, whereas R131K substitutions and truncated *rdxA* were also detected in resistant strains. In contrast, the A68N mutation was found in susceptible isolates.

Resistance levels were associated with specific mutations. High‐level clarithromycin resistance (MIC > 8 *μ* *g*/mL) was associated with A2143G (*n* = 2) or dual A2143G + A2142G mutations (*n* = 1). Low‐level resistance was observed in isolates with no detected mutations. For levofloxacin, high‐level resistance (MIC > 2 *μ* *g*/mL) was observed in isolates harboring D91N and D91G, whereas low‐level resistance was associated with N87K. Metronidazole high‐level resistance (MIC > 32 *μ* *g*/mL) was associated with *rdxA* mutations including D59N, protein truncations, and the 200‐bp deletion. These findings suggest an association between certain mutations and elevated MIC values.

Mutation patterns varied across clinical subgroups. The A2143G mutation was mainly observed in PUD (2/3 cases) and was absent as a sole mutation in NUD and GC. A2142G occurred in both PUD and NUD, whereas the dual A2143G + A2142G mutation was observed in the only GC case. Notably, the three isolates lacking *23S rRNA* mutations were from the NUD group. *gyrA* mutations showed less distinct distribution, though D91N and D91G were observed in PUD, N87K in NUD, and D91N was identified in the GC case. *rdxA* mutations, including the 200‐bp deletion, were found across all groups (Table S1).

**Figure 3:**
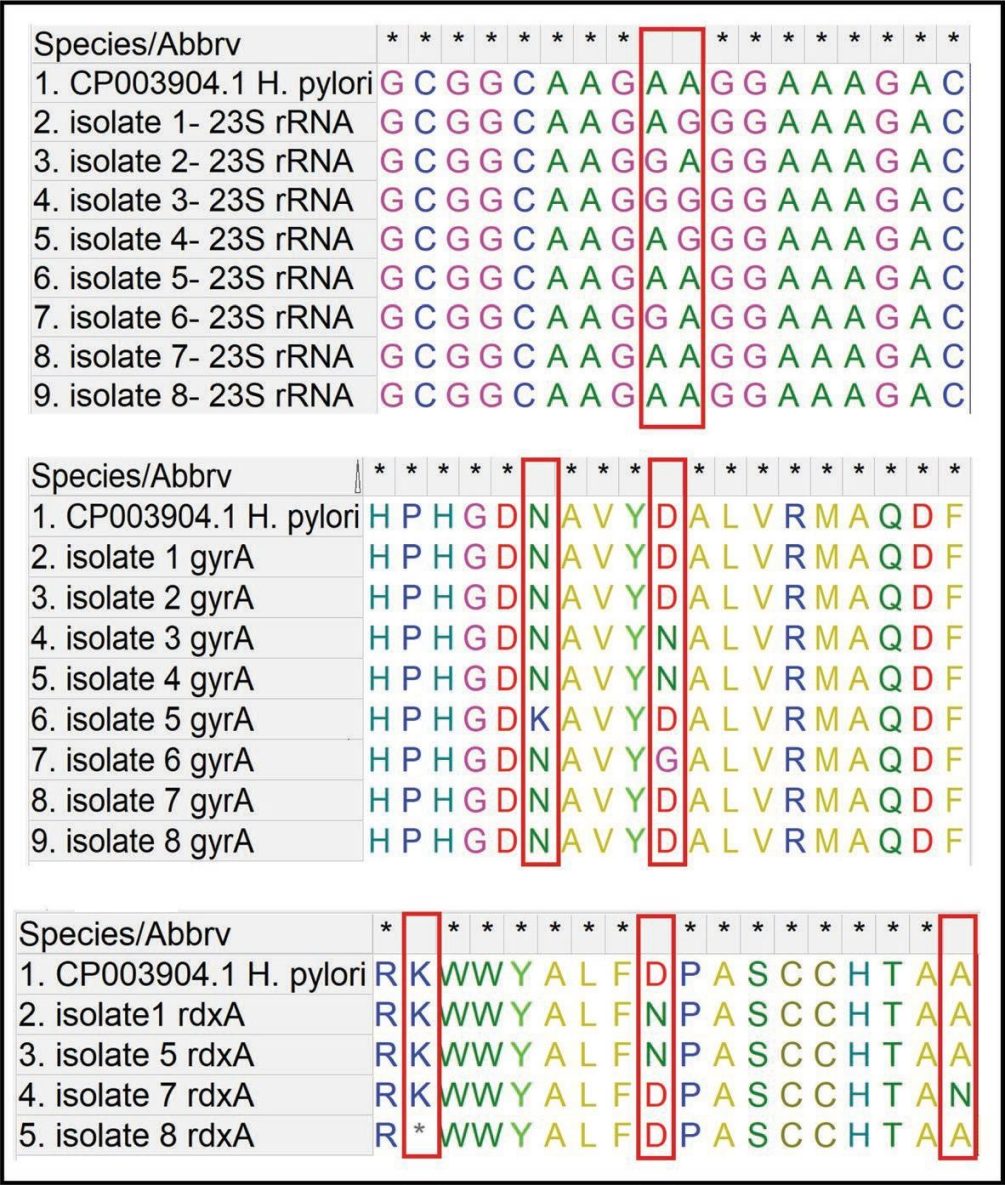
MEGA 11 alignment of *23S rRNA*, *gyrA*, and *rdxA* gene sequences from *H. pylori* clinical isolates relative to reference strain 26695, highlighting mutation hotspots (red boxes).

MEGA 11 alignments illustrated the spectrum of mutations across isolates (Figure 3). Of the eight sequenced isolates, 75% harbored combined mutations involving multiple resistance genes, with triple mutations being most frequent (37.5%). Only two isolates (25%) showed a single *rdxA* mutation (Figure S3). Combined mutations were more frequently observed in isolates from patients with recurrent infection and in coccoid (66.7%) or coccobacillary (33.3%) forms, whereas single mutations were generally observed in bacillary forms. These observations suggest that combined genetic alterations may be associated with persistent infection and changes in bacterial morphology, which pointing to challenges in treatment and eradication, particularly in cases with advanced pathology.

For the clinical implications of our findings, the phenotypic and genotypic characteristics of the sequenced *H. pylori* isolates are summarized in Table 5. The observed resistance profiles highlight the potential value of individualized, susceptibility‐guided treatment strategies. In particular, bismuth‐based regimens, rifabutin‐containing salvage therapies, potassium‐competitive acid blocker (PCAB)–based therapies, and selected levofloxacin‐containing combinations may represent potential alternatives for isolates exhibiting multidrug resistance. However, these therapeutic considerations are based on a limited number of sequenced isolates and should be interpreted with caution. Further studies involving larger numbers of isolates are required to validate the clinical utility of genotype‐guided treatment recommendations.

**Table 5:** Clinical profile and potential treatment approaches for patients harboring selected *H. pylori* isolates.

Isolate code	Tissue form	Resistance pattern	Molecular findings	Clinical profile	Potential treatment approaches/tailored regimens
			(Gene: mutation)		
**HP1**	Coccobacilli	Dual (CLR.+MTZ.)	*—23S rRNA:* A2143G	Male patient < 45 years with (PUD), a single recurrence, mixed gastric microbiota detected in culture, a treatment history of standard triple therapy, and no comorbidities.	***—*** Modified bismuth‐based quadruple therapy (PPI + bismuth + tetracycline + amoxicillin), to avoid CLR./MTZ.
			*—gyrA:* no mutation		
			*—rdxA:* D59N		
			***—*** Levofloxacin‐based triple therapy		
**HP2**	Coccoid	Dual (CLR.+MTZ.)	*—23S rRNA:* A2142G	Female patient < 45 years with (NUD), multiple recurrences, mixed gastric microbiota detected in culture, a treatment history including multiple courses of standard triple therapy as well as bismuth quadruple therapy, and comorbid liver disease.	***—*** Levofloxacin‐based triple therapy
			***—**gyrA:* no mutation		***—*** PCAB dual regimen
			***—**rdxA:* 200‐bp deletion		

**HP3**	Coccoid	MDR (CLR.+LEV.+MTZ.)	***—**23S rRNA:* A2142G + A2143G	Male patient > 45 years with (GC), multiple recurrences, mixed gastric microbiota detected in culture, a treatment history including multiple courses of standard triple therapy and LOAD therapy and comorbid autoimmune disease.	***—*** PCAB dual regimen
			***—**gyrA:* D91N		***—*** Bismuth − based quadruple therapy + culture − guided salvage therapy.
			***—**rdxA:* 200‐bp deletion		

**HP4**	Coccoid	MDR (CLR.+LEV.+MTZ.)	***—**23S rRNA:* A2143G	Female patient < 45 years with (PUD), a single recurrence, mixed gastric microbiota detected in culture, a treatment history of standard triple therapy, and no comorbidities.	***—*** PCAB dual regimen
			***—**gyrA:* D91N		***—*** Bismuth‐based quadruple therapy + culture‐guided salvage therapy.
			***—**rdxA:* 200‐bp deletion		

**HP5**	Coccobacilli	Dual (LEV.+MTZ.)	***—**23S rRNA:* no mutation	Female patient > 45 years with (NUD), a single recurrence, mixed gastric microbiota detected in culture, a treatment history of bismuth quadruple therapy, and comorbid liver disease.	***—*** PCAB‐based regimens
			***—**gyrA:* N87K		***—*** Peptic care therapy
			***—**rdxA:* D59N		

**HP6**	Coccoid	Dual (LEV.+MTZ.)	***—**23S rRNA:* A2142G	Male patient > 45 years with (PUD), multiple recurrences, mixed gastric microbiota detected in culture, a treatment history including multiple courses of standard triple therapy and LOAD therapy, and comorbid autoimmune disease.	***—*** PCAB‐based regimens
			***—**gyrA:* D91G		***—*** Peptic care therapy
			***—**rdxA:* R131K		

**HP7**	Bacilli	Mono (CLR.)	***—**23S rRNA:* no mutation	Male patient < 45 years with (NUD), no history of recurrence, a pure *H. pylori* culture, and comorbid hypertension.	***—*** Modified standard triple therapy (PPI + amoxicillin + metronidazole) could still be effective, to avoid CLR.
			***—**gyrA:* no mutation		
			***—**rdxA:* A68N		***—*** Modified bismuth‐based quadruple therapy (PPI + bismuth + tetracycline + amoxicillin), to avoid CLR.

**HP8**	Bacilli	Mono (MTZ.)	***—**23S rRNA:* no mutation	Female patient > 45 years with (NUD), no history of recurrence, mixed gastric microbiota detected in culture, and comorbid hypertension and livre disease.	***—*** Levofloxacin‐based triple therapy
			***—**gyrA:* no mutation		***—*** PCAB‐based regimens
			***—**rdxA*: truncated *rdxA*		***—*** Peptic care therapy

*Note:* The presence of the 200‐bp *rdxA* deletion was determined by PCR analysis, whereas sequencing was performed to identify point mutations in resistance‐associated genes. Standard triple therapy: (PPI + clarithromycin + metronidazole), bismuth‐based quadruple therapy: (bismuth + metronidazole + tetracycline + PPI), LOAD therapy: (levofloxacin + omeprazole + Alinia + doxycycline). Levofloxacin‐based triple therapy: (PPI + amoxicillin + levofloxacin), PCAB dual regimen: (vonoprazan + amoxicillin), PCAB based regimens: (vonoprazan + amoxicillin + clarithromycin + clarithromycin), peptic care therapy: (clarithromycin + tinidazole + omeprazole). Rifabutin triple regimen: (omeprazole + amoxicillin + rifabutin) if available can be used as a rescue therapy in treatment of cases with HP2,3,4,5,6 as a line of culture‐based treatment to avoid resistance development.

Abbreviations: CLR, clarithromycin; LEV., levofloxacin; MDR, multidrug resistant; MTZ, metronidazole; PCAB, potassium‐competitive acid blocker; PPI, proton pump inhibitor.

## 4. Discussion

*H*. *pylori* is a distinctive gastric pathogen increasingly recognized for its growing resistance to antimicrobial therapies [1]. In this study, endoscopic assessment of 240 dyspeptic patients revealed that NUD was the most frequent diagnosis followed by PUD and finally GC in alignment with data revealed from several Arab and African countries, where gastritis and NUD predominate, and GC remains relatively uncommon [23] [24]. In contrast, other regions report disparities in disease prevalence and clinical outcomes reflecting variations in *H. pylori* strain virulence plus differences in diagnostic and therapeutic practices [25].

Currently, no single diagnostic modality is universally accepted as the gold standard for detecting *H. pylori* in gastric tissue [18]. In this study, we assessed three commonly utilized methods (RUT, histopathology, and culture). Our detection rates were comparable to those reported by Majaliwa et al. [26]. Although culture remains the most specific method and indispensable for antimicrobial susceptibility testing, it is prone to false negatives, especially in samples containing coccoid forms [9]. Biopsy handling issues such as sharing with histopathology, formalin exposure or delays, and the fastidious microaerophilic requirements of *H. pylori* could likely reduce bacterial viability and underestimate culture‐positive cases. In contrast, histopathology and molecular techniques, such as PCR, demonstrate greater sensitivity, and so employing a combination of diagnostic approaches enhances detection accuracy and reliability [27].

Microscopically, among 129 histopathology‐positive cases, coccobacillary form was the most frequently observed morphology (44.9%), followed by the bacillary (34.8%) and coccoid forms (20.3%). Notably, the coccoid form was identified in a GC case and in most of the PUD cases, aligning with findings by Zhong et al. [28], which reported an association between coccoid transformation with advanced gastric pathology. These nonspiral forms are known to be viable but nonculturable (VBNC), which may explain culture failures under antibiotic exposure or environmental stress. In addition, mixed microbial colonization was most prominent among PUD patients (80.9%), and to a lesser extent in NUD cases (50.3%), particularly those with atrophic gastritis. This pattern supports the hypothesis that non‐*H. pylori* gastric microbiota may play a role in disease progression, especially under conditions of reduced gastric acidity and enhanced antigenic stimulation, as previously proposed by Verma et al. [29].

Although susceptibility‐guided therapy has been shown to improve eradication rates, it is not yet routinely applied in Egypt [1]. In this study, we assessed disk diffusion as a practical alternative, showing high accuracy for levofloxacin (97%) and acceptable concordance for clarithromycin and metronidazole (≈85%) when compared with the gold‐standard agar dilution method. Several studies have demonstrated satisfactory susceptibility agreement between the disk diffusion method and agar dilution or E‐test methods for testing *H. pylori* resistance to CLR, LEV, and MTZ [30]. This approach is further supported by the lower cost, wider availability, and greater applicability of disk diffusion in developing countries [18].

Nevertheless, it should be noted that standardized interpretative criteria for the disk diffusion method in *H. pylori* are still not universally established. Reference methods such as agar dilution and E‐test remain the recommended approaches for antimicrobial susceptibility testing. In particular, susceptibility testing for metronidazole may show variable results with disk diffusion due to methodological limitations. Therefore, although our findings demonstrated acceptable concordance with the agar dilution method, the results should be interpreted cautiously [18].

Using disk diffusion versus agar dilution, we observed notable resistance to metronidazole (86.4% vs. 72.8%) and clarithromycin (63% vs. 23.5%), whereas levofloxacin maintained high susceptibility across both methods (87.7% vs. 90.1%). These findings were aligned with reports from Egypt [31], other Arab countries [32], and Europe [33]. However, variations in resistance rates reported by other studies [34, 35] may reflect the influence of regional differences in antibiotic usage, diagnostic approaches, socioeconomic and sanitation levels, factors that collectively may influence reinfection and treatment success; especially with widespread drug use further underscores the need for precise susceptibility testing before initiating therapy.

In our study, infection recurrence was significantly associated with dual and triple resistance patterns, This finding suggests that *H. pylori* resistance may be influenced by prior antibiotic exposure and reinfection rather than host‐related factors, aligning with Azadbakht et al. [36] and Zhou et al. [37] who showed that repeated or incomplete treatments promote resistant strains, also Vu et al. [3] linked recurrence to reduced antibiotic efficacy and high resistance.

High levels of antibiotic resistance are associated with reduced effectiveness of commonly used drugs, potentially limiting their suitability as first‐line therapy, whereas lower resistance levels may still allow cautious use as rescue therapy. The coccoid transition has been reported to be associated with stress‐response gene regulation, peptidoglycan modification, and immune evasion, representing a VBNC state that may contribute to bacterial persistence and increased tolerance to antibiotics [12]. In addition, coccoid morphology may be associated with immune evasion and potential treatment failure, which may complicate management, as eradication may require longer treatment durations, combination regimens, and close follow‐up, as suggested in previous studies [38].

Resistance in *H. pylori* is predominantly associated with point mutations [4]. In this work, PCR analysis revealed a 200‐bp deletion in the *rdxA* gene in 47.5% of metronidazole‐resistant isolates. This truncated amplicon (650 bp) may reflect gene inactivation and was found to be associated with higher levels of resistance. The categorization of high‐ and low‐level metronidazole resistance was determined according to De Francesco et al. [39] study, whereas primary susceptibility interpretation was based on EUCAST 2022 criteria. The global variability in the prevalence of this deletion underlines the potential importance for regional molecular surveillance to guide tailored treatment strategies [40].

We observed *rdxA* gene 200‐bp deletion mutation in the GC patient isolated *H. pylori* and it was associated with a higher level of metronidazole resistance. Other mutations, such as D59N and R131K, were identified in NUD and PUD isolates but were associated with variable resistance levels, consistent with previous findings [41]. These findings suggest that MIC testing may be important for resistance profiling and guiding treatment decisions [42].

Five out of eight sequenced isolates harbored clarithromycin mutations, with A2143G and A2142G being the most frequently detected among PUD cases. The combined A2143G + A2142G mutation detected in GC case was observed to be associated with high‐level resistance, as A2143G alone, whereas A2142G was observed in isolates with lower resistance levels. Clarithromycin resistance levels break points were also determined according to De Francesco et al. [39] study. These observations were aligned with both regional and international studies [41] and [43], may support the potential value of integrating molecular and phenotypic testing.

Although newer mutations in the *23S rRNA* gene, such as A2115G, A2144T, and G2141A, have been reported in some studies, these mutations were not detected in any of the eight isolates sequenced in our study. We observed that A2142G and A2143G mutations were the predominant mutations associated with clarithromycin resistance. A2142C mutation was not detected in any of the sequenced isolates. Future studies with larger sample sizes may help determine the prevalence of less frequent mutations in the population.

All four levofloxacin‐resistant isolates on sequencing carried *gyrA* mutations: D91N (*n* = 2), D91G (*n* = 1), and N87K (*n* = 1). The D91N mutation, which was observed with varying levels of resistance, was observed in PUD and GC cases. Levofloxacin high and low resistance levels were determined according to López‐Gasca et al. [4] study. In contrast, N87K and wild‐type isolates were found in NUD cases, which may reflect earlier stages of infection. These findings were consistent with previous reports [4] and [5]. Molecular analysis in our study was limited to *gyrA* mutations, as they represent the primary and most frequently reported mechanism of fluoroquinolone resistance in *H. pylori.*

The A2143G mutation in the *23S rRNA* gene was observed more frequently in clarithromycin‐resistant isolates compared with A2142G, consistent with previous reports [44, 45]. Similarly, *gyrA* mutations at Codons 87 and 91 were observed only in levofloxacin‐resistant isolates, in agreement with prior studies [46]. Regarding metronidazole resistance, *rdxA* mutations, including D59N, deletions, and truncated forms, were mainly observed in resistant strains, whereas the presence of A68N in susceptible isolates suggests that some *rdxA* substitutions may represent neutral polymorphisms rather than true resistance determinants [47]. Though *frxA* mutations are known contributors to metronidazole resistance, this gene was not analyzed in the current study and may explain resistance in some other isolates.

Although these findings observed a noticeable association between specific mutations in *23S rRNA*, *gyrA*, and *rdxA* genes and resistance to clarithromycin, levofloxacin, and metronidazole, respectively, the relatively limited number of sequenced isolates may influence the strength of the observed associations and restrict the ability to generalize these findings. Further studies involving larger numbers of sequenced isolates are needed to better clarify the potential role of these mutations and their relevance to antibiotic resistance.

Furthermore, combined resistance mutations involving *23S rRNA*, *gyrA*, and *rdxA* were detected in 75% of sequenced isolates, consistent with recent studies [48]. Such multilocus resistance has been observed in cases of treatment failure and recurrence, highlighting the potential value of advanced tools such as whole‐genome sequencing (WGS) for supporting personalized therapy and informing strategies aimed at improving eradication outcomes [6].

The treatment implications of our findings are clinically relevant. Bacillary isolates, which predominantly exhibited mono‐resistance, may remain amenable to standard clarithromycin‐based triple therapy. In contrast, coccoid and coccobacillary forms, particularly those associated with mixed gastric microbiota, demonstrated dual or multidrug resistance linked to mutations in *23S rRNA*, *gyrA*, and *rdxA*, highlighting the potential value of susceptibility‐guided and genotype‐informed treatment strategies. For such resistant strains, alternative eradication approaches, including bismuth‐containing quadruple therapy, rifabutin‐based salvage regimens, and other optimized treatment protocols (e.g., sequential, concomitant, hybrid, reverse hybrid, or LOAD therapies), may be considered. As the effectiveness of these regimens is influenced by local antimicrobial resistance patterns, treatment selection should be guided whenever possible by phenotypic susceptibility testing, molecular resistance profiling, and regional resistance data to maximize eradication success [22].

## 5. Conclusion and Recommendations

This study highlights a relatively high prevalence of antimicrobial resistance in *H. pylori*, particularly to clarithromycin and metronidazole, which appears to be associated with mutations in the *23S rRNA* and *rdxA* genes, respectively. Given the limitations of conventional diagnostics and genetic challenges, coccoid morphology, microbiota mixed infection, and high recurrence rates may represent potential indicators of impaired therapeutic response.

Our findings suggest a possible concordance between genotype and phenotype and support the integration of molecular assays with phenotypic testing. However, considering the limited number of sequenced isolates, these observations should be interpreted with caution. Future studies incorporating advanced approaches such as WGS may further elucidate the interplay of multiple resistance determinants, uncover novel mechanisms, and could provide insights into more effective and individualized eradication strategies in regions with high resistance rates.

### 5.1. Limitation

The lack of financial support limited the extension of PCR assays and sequencing to a larger number of isolates and prevented the application of WGS. Consequently, only eight isolates were selected for sequencing to best represent the criteria presented in the results. This limitation, combined with potential overlap in clinical data between patients, restricts the generalizability of the findings, despite repeated observations across some patients. The small number of sequenced isolates limits the ability to perform robust statistical analyses.

**Nomenclature**
CLR: clarithromycin
EUCAST: European Committee on Antimicrobial Susceptibility Testing
GC: gastric cancer
H&E: hematoxylin–eosin
LEV: levofloxacin
LOAD: $\begin{array}{c} \text{levofloxacin} \end{array}$ + omeprazole + alinia (nitazoxanid)e + doxycycline
MDR: multidrug resistant
MIC: minimum inhibitory concentration
MTZ: metronidazole
NPV: negative predictive value
NUD: nonulcer dyspepsia
PCAB: potassium‐competitive acid blocker
PCR: polymerase chain reaction
PPI: proton pump inhibitor
PPV: positive predictive value
PUD: peptic ulcer disease
QRDR: quinolone resistance‐determining region
rRNA: ribosomal ribonucleic acid
RUT: rapid urease test
WGS: whole‐genome sequencing

## Author Contributions

**Amal F. Makled:** conceptualization (lead), formal analysis (equal), supervision (lead), visualization (equal), and writing of the original draft (equal). **Hany A. Abdelaziz**: consultant gastroenterologist shared in gastric biopsies expert collection and endoscopic data analysis and interpretation. **Dina M. Allam**: histopathological slides assessment, interpretation, and writing and editing. **Mona F. Salama**: performed the methodology. **Nashwa N. Khamis**: practical methodology (equal) and investigation (equal). **Asmaa S. Sleem**: methodology (equal), supervision (equal); writing, editing, and data analysis.

## Funding

No funding was received for this manuscript.

## Disclosure

Each author listed in the manuscript approved the submission of this version of the manuscript and takes full responsibility for it.

## Ethics Statement

An informed written consent was obtained from all participants before enrolling in this study and the study protocol was approved by the Local Ethical Committee of Faculty of Medicine, Menoufia University (IRB: 7/2023MICR20). The study was conducted following good clinical practice and the Declaration of Helsinki.

## Consent

The authors have nothing to report.

## Conflicts of Interest

The authors declare no conflicts of interest.

## Supporting information


**Supporting Information** Additional supporting information can be found online in the Supporting Information section. Figure S1: Endoscopic findings in the study population, highlighting NUD, PUD, and GC groups′ main mucosal lesions. Figure S2: Distribution of gastric bacterial isolates across endoscopic groups (NUD, PUD, and GC). Table S1: PCR and sequencing results of the studied genes in selected *H. pylori* isolates in relation to endoscopic groups. Figure S3: Mutation patterns of sequenced isolates.

## Data Availability

The partial *23S rRNA* gene sequences of the eight sequenced *H. pylori* isolates were deposited in the GenBank database under the Accession Numbers PX249695–PX249702. The partial coding sequence (CDS) of the *gyrA* gene of the eight sequenced *H. pylori* isolates was deposited in GenBank under the Accession Numbers PX275437–PX275444. The partial coding sequence (CDS) of the *rdxA* gene of the five sequenced *H. pylori* isolates was deposited in GenBank under the Accession Numbers PX275445–PX275449. Other data used to support the findings of this study are available from the corresponding author upon reasonable request.
