# Supplementary material for: Phenotypic and Genotypic Characterization of Antimicrobial Resistance in Helicobacter pylori: Association of 23S rRNA, gyrA, and rdxA Gene Mutations With Resistance Patterns
Source: Int J Microbiol. 2026 Jul 6;2026:6456370. doi: 10.1155/ijm/6456370 (PMC13334456; doi:10.1155/ijm/6456370)
Supplement: Supplementary file 1 — Supporting Information Additional supporting information can be found online in the Supporting Information section. Figure S1: Endoscopic findings in the study population, highlighting NUD, PUD, and GC groups′ main mucosal lesions. Figure S2: Distribution of gastric bacterial isolates across endoscopic groups (NUD, PUD, and GC). Table S1: PCR and sequencing results of the studied genes in selected H. pylori isolates in relation to endoscopic groups. Figure S3: Mutation patterns of sequenced isolates. [file IJM-2026-6456370-s001.docx]

**Supplementary Tables and Figures**

**
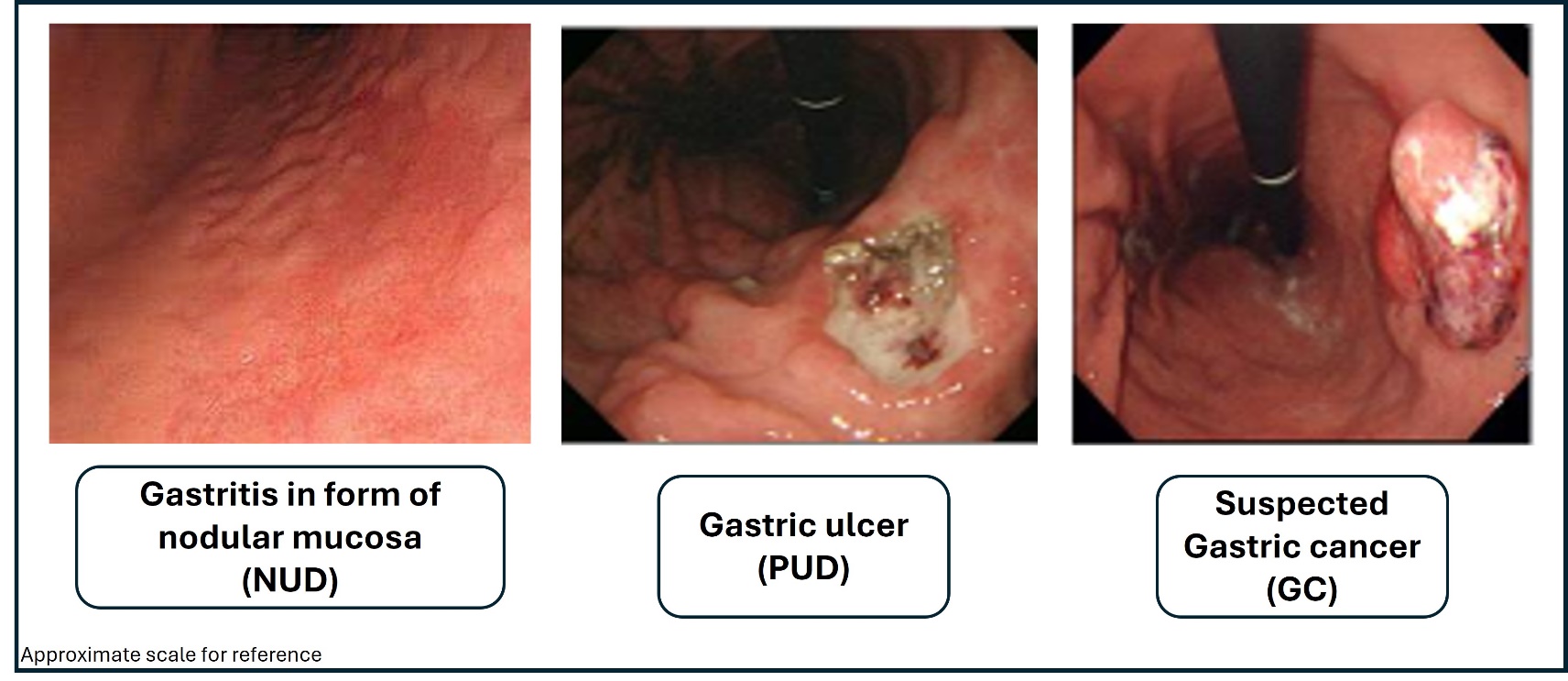
**

**Figure (1- S):** **Endoscopic findings in the study population, highlighting NUD, PUD and GC groups’ main mucosal lesions.**

**Figure (2- S):** **Distribution of gastric bacterial isolates across endoscopic groups (NUD, PUD, GC):**

**Table (1- S):** **PCR and sequencing results of the studied genes in selected *H. pylori* isolates in relation to endoscopic groups:**

| Antibiotic / gene | Endoscopic groups | | | | | |
| --- | --- | --- | --- | --- | --- | --- |
|  | **NUD**  **(N=4)** | | **PUD**  **(N=3)** | | **GC**  **(N=1)** | |
|  | Mutation | N (%) | Mutation | N (%) | Mutation | N (%) |
| Clarithromycin/ *23S rRNA* gene | **A2142G** | 1 (25) | **A2143G** | 2 (66.7) | **A2142G + A2143G** | 1 (100) |
|  | **No mutation** | 3 (75) | **A2142G** | 1 (33.3) |  |  |
| Levofloxacin/ *gyrA* gene | **N87K** | 1 (25) | **D91N** | 1 (33.3) | **D91N** | 1 (100) |
|  | **No mutation** | 3 (75) | **D91G** | 1 (33.3) |  |  |
|  |  |  | **No mutation** | 1 (33.3) |  |  |
| Metronidazole/ *rdxA* gene | **200 bp deletion** | 1 (25) | **200 bp deletion** | 1 (33.3) | **200 bp deletion** | 1 (100) |
|  | **D59N** | 1 (25) | **D59N** | 1(33.3) |  |  |
|  | **Truncated protein** | 1 (25) | **R131K** | 1(33.3) |  |  |
|  | **A68N** | 1 (25) |  |  |  |  |

- **N: Number of patients**

**Figure (3- S):** **mutation patterns of sequenced isolates:**
